# Research into the intervention effect of folic acid on arsenic-induced heart abnormalities in fetal rats during the periconception period

**DOI:** 10.1186/s12872-020-01418-z

**Published:** 2020-03-17

**Authors:** Lin Na, Bai Q, Zhao Xiumei, Zhuang Lingzi, He Deqin, Zhuang Xuanxuan, Guo Huanhuan, Lin Yuan, Chen Xiujuan

**Affiliations:** 1 Department of the Prenatal Diagnosis Center, Fujian Maternity and Child Health Hospital, Affiliated Hospital of Fujian Medical University, Fujian Key Laboratory for Prenatal Diagnosis and Birth Defect, Fuzhou, China; 2 Department of Obstetrics and Gynecology, Fujian Maternity and Child Health Hospital, Affiliated Hospital of Fujian Medical University, Daoshan Road 18, Gulou District, Fuzhou, 350005 Fujian China

**Keywords:** Congenital heart disease, Folic acid, Arsenic, Histone H3K9, Mef2C

## Abstract

**Background:**

The incidence of CHD is the highest among birth defects and is increasing year to year. CHD seriously harms the health of infants and young children and presents a large economic burden to families and society. The pathogenesis of CHD and preventive measures are the focus of current research. Our research aimed to explore the intervention effect of folic acid on heart abnormalities resulting from sodium arsenic (NaAsO_2_) exposure during the periconception period.

**Methods:**

Sixty 35-day-old female SD rats were randomly divided into 5 groups with 12 rats in each group. Group A was the control group. The rats were given distilled water and ordinary chow. The rats in group B were given distilled water containing 75 mg/L NaAsO_2_ and ordinary chow. The rats in groups C, D, and E were given distilled water containing 75 mg/L NaAsO_2_ and chow containing 0.53 mg/kg, 5.3 mg/kg, and 10.6 mg/kg folic acid, respectively. The general condition of the embryos and the histopathology of the embryonic hearts were examined. The acetylation levels of histone H3K9 in heart tissues and the expression levels of Mef2C (which is related to heart development) were observed.

**Results:**

The embryo weight and placental weight of groups B-E were significantly lower than those of group A (*P* < 0.05). The heart malformation rate of the fetal rats in groups B-E was significantly higher than that of the fetal rats in group A (*P* < 0.05). We found that the level of H3K9 acetylation in fetal rat cardiomyocytes in groups B-E was significantly higher than that in group A (*P* < 0.05) and that the level of H3K9 acetylation in groups C-E was lower than that in group B (*P* < 0.05). The mRNA level of Mef2C in fetal rat cardiomyocytes in group B-E was significantly higher than that in group A (*P* < 0.05), and the mRNA level of Mef2C in groups C-E was significantly lower than that in group B (*P* < 0.05).

**Conclusion:**

Supplementation with folic acid during the periconception period can interfere with the toxic effects of arsenic on the heart. The mechanism may be that lowering the acetylation levels of histone H3K9 in heart tissues leads to decreased expression levels of Mef2C, which may play a protective role in heart development in fetal rats.

## Background

Congenital heart disease (CHD) is an abnormality in morphological structure and functional metabolism caused by abnormal development of the heart and large blood vessels during the embryonic period or a congenital deformity in which the orifices that are present during the fetal period and are used for blood circulation remain open after birth. Common types of CHD include atrial septal defect, ventricular septal defect, tetralogy of Fallot and complete transposition of the main arteries. In China, approximately 900,000 new cases of birth defects occur each year, and the incidence rate is as high as 5.6%. CHD has the highest incidence of all birth defects, and the incidence is increasing year to year [1]. CHD seriously harms the health of infants and young children and presents a large economic burden to families and society. Therefore, it is of great significance to explore the pathogenesis of and preventive measures for CHD.

At present, the pathogenesis of CHD is not clear, and it is mainly thought to be caused by environmental factors, genetic factors and the interaction between the two [2, 3]. Relevant data show that only 40% of CHD cases can be explained by genetic abnormalities and chromosomal abnormalities and that the majority are caused by the combined actions of genetic factors and environmental factors [4]. At the early stage of development, fetuses are highly sensitive to environmental factors. Changes in the external environment, such as a lack of essential nutrients, exposure to toxic substances, infection with viruses, and unhealthy lifestyles, such as smoking and drinking during pregnancy, can affect the formation and development of the embryonic heart. The development of the heart is delicate and complex, requiring accurate expression of related genes in different locations and at different times. Any small interference can affect the normal formation and development of the heart, which leads to the occurrence of CHD [5]. There is increasing evidence that in addition to genetic factors, epigenetic inheritance also plays a key role in the precise expression of genes involved in cardiac development. The main components of epigenetics include DNA methylation, histone modification, noncoding RNA regulation and chromatin remodeling. Among histone modifications, the earliest known transcription-related acetylation modification plays an important role in regulating gene transcription. The acetylation of histone H3 lysine 9 (H3K9) plays an important role in gene transcription and expression [6]. Myocyte enhancer factor-2C (Mef2C) is a core transcription factor during cardiac development, and the abnormal expression of Mef2C can cause abnormal cardiac development and lead to the occurrence of congenital heart disease. A previous study found that [7] histone acetylation may be an important factor that regulates the expression of the Mef2C gene. Arsenic is a toxic metal that is widely found in nature. Inorganic arsenic can interfere with DNA methylation, histone modification, and noncoding RNA expression [8, 9]. Our preliminary study showed as exposure to NaAsO_2_ increases, the incidence of CHD in fetal rats increases. At the same time, arsenic exposure in fetal rats increases the acetylation levels of histone H3K9 in the myocardium and the expression levels of Mef2C compared to those in the control group. This suggests that arsenic may induce cardiac malformation through epigenetic action.

Folic acid is an essential nutrient for the human body. Folic acid deficiency or the inhibition of biological activities can lead to birth defects such as neural tube defects, CHD, cleft lip and palate deformity [10, 11]. If folic acid is taken during pregnancy, it can effectively reduce the incidence of premature birth and low birth weight [12, 13]. At present, research on the mechanism by which folic acid prevents birth defects mainly focuses on folate deficiency and one-carbon metabolism disorders, and there are few reports on epigenetic mechanisms. Studies have shown that folate metabolism can increase the level of NAD-dependent histone deacetylase sirtuins (SIRTs) by increasing the level of nicotinamide adenine dinucleotide coenzyme (NAD) [14], resulting in changes in histone acetylation levels and thus affecting cell survival, aging and apoptosis. In this study, female rats exposed to arsenic during the periconception period were fed chow containing folic acid to observe the development of the fetal heart, and the effects of different doses of folic acid on developmental toxicity in fetal rats induced by arsenic were investigated. The possible mechanism of the intervention was explored to provide a theoretical basis for supplementation with folic acid during pregnancy to prevent CHD.

## Methods

### Animals

Healthy, clean female SD rats (35 days old) were purchased from the Animal Center of Fujian Medical University (Fuzhou, China). Animal care and experimental proceedings were carried out according to guidelines established by the Fujian Medical University Animal Care Commission and following the approval of the Fujian Medical University Laboratory Animal Welfare&Ethics Committee. The ambient temperature was 20 ± 2 °C, the relative humidity was approximately 55%, and the illumination time was 12 ± 3 h. Water was changed three times a week, and the animal houses and animal cages were cleaned regularly.

### Grouping and arsenic exposure

Sixty 35-day-old female SD rats were used. The rats were fed for 7 days to allow adaptation to the environment and were randomly divided into 5 groups with 12 rats in each group according to body weight. Group A was given distilled water. Group B was given distilled water containing 75 mg/L NaAsO_2_. Groups C, D, and E were given distilled water containing 75 mg/L NaAsO_2_ and chow containing 0.53 mg/kg, 5.3 mg/kg, and 10.6 mg/kg folic acid, respectively. The protocol continued for 6 weeks. After that, the female SD rats were placed in cages with male SD rats at a ratio of 2:1 overnight. The next morning, the presence of vaginal plugs was used to determine whether the female rats were pregnant. Female rats with vaginal plugs, were considered to be in gestational day zero. The pregnant female rats received the same feeding protocol they received after grouping until they were 16 days pregnant. Then, the embryos were removed by cesarean section, and the rats were euthanized via cardiac puncture after cesarean section under 1–2% isoflurane anesthesia. If a female rat was observed to have a vaginal plug but had no embryos on the 16th day it was considered to have had a miscarriage.

### General condition and pathological observation

On the 16th day, caesarean section was carried out under 1–2% isoflurane anesthesia. The number of rats that had miscarriages was recorded, and the live fetal rats and placentas were weighed. All the fetal rats were euthanized via decapitation with surgical scissors under 1–2% isoflurane anesthesia after caesarean section. Three fetal rats from each litter were randomly selected, and the hearts and both lungs (the latter as a reference tissue) were collected and placed them in 10% neutral buffered formalin for 24 h. Then, the tissues were dehydrated, embedded in paraffin, cut continuously at a thickness of 7 μm, stained with hematoxylin-eosin (HE), and sealed. Morphological changes in the fetal rat hearts were observed under a light microscope.

### Real-time PCR detection of the relative expression of Mef2C mRNA

Total RNA was extracted from fetal rat heart tissues using TRIzol reagent (Invitrogen, USA) according to the protocol. Two micrograms of total RNA was used for reverse transcription with iScript RT Supermix (Bio-Rad, USA) according to the instructions. The reaction conditions were 65 °C for 5 min, 25 °C for 5 min, 42 °C for 60 min, and 70 °C for 5 min. Primers were synthesized by Shanghai Biotech Bioengineering Co., Ltd. Glyceraldehyde 3-phosphate dehydrogenase (GAPDH) was used as an internal standard. The primer sequences for GAPDH were 5′-TGATTCTACCCACGGCAAGT-3′ and 5′-AGCATCACCCCATTTGATGT-3′. The primer sequences for Mef2C were 5′-GCACCTACATAACATGCCGC-3′ and 5′-GCTTTGAGTAGAAGGCAGGGA-3′. The PCR conditions were as follows: UDG pretreatment at 50 °C for 2 min, initial denaturation at 95 °C for 10 min, renaturation at 95 °C for 15 s, annealing at 55 °C for 30 s, and 72 °C for 32 s for 42 cycles. The relative expression of the target gene Mef2C was determined according to the comparative threshold method described by Livak and Schttgen. The relative expression level of the target gene was 2^-ΔΔCt^.

### Detection of the relative protein expression of acetylated histone H3K9 in the myocardium via Western blot analysis

Heart tissues (20 mg) were thoroughly homogenized with a plastic grinding rod at 4 °C. Nuclear protein was extracted according to the instructions and quantified by a BCA assay. The gels were 12% separation and 5% spacer gels. The same amount of protein from different samples was mixed well with 5× SDS protein loading buffer at a ratio of 4:1 and denatured at 100 °C for 5–10 min. Then, the proteins were loaded onto SDS-polyacrylamide gels for electrophoresis. The proteins were run on the spacer gel at 80 V and on the separation gel at 100 V and blotted onto polyvinylidene fluoride (PVDF) membranes. The PVDF membranes were blocked in 5% bovine serum albumin for 60 min at room temperature. The blocked PVDF membranes were placed in diluted primary antibody (H3K9, Abcam, UK; Lamin B1, Boster, China) overnight at 4 °C, washed, placed in diluted secondary antibody (HRP-linked antibody, Merck, Germany), and incubated for 1 h at room temperature. The PVDF membranes were placed in the reaction solution of a chemiluminescence kit, incubated for 2 min at room temperature and exposed to film. The film was scanned and saved, and grayscale analysis was performed using Quantity One software to calculate the gray value of Mef2C/Lamin B1 for each group.

### Statistical analysis

Data analysis was performed using SPSS 18.0 statistical software. After inspection, the data were found to be normally distributed with equal variance, and the data are expressed as *x* ± *s*. The data were analyzed using analysis of variance (ANOVA), and comparisons among groups were analyzed by the Student-Newman-Keuls (SNK) test. Quantitative data were statistically analyzed using the Chi-square test and Fisher’s exact probability test. *P* < 0.05 indicated a significant difference.

## Results

### General condition

The number of pregnant rats in groups A, B, C, D, and E was 10, 11, 10, 10, and 10, respectively. The number of miscarriages in groups A, B, C, D, and E was 1, 1, 0, 1, and 0, respectively. The embryo weight and placental weight of groups B, C, D and E were significantly lower than those of group A (*P* < 0.05). The embryo weight and placental weight of groups D and E were significantly increased compared with those of group B (*P* < 0.05) (Table 1).

**Table 1:** Embryogenic toxicity of NaAsO2 and protective effect of folate

Groups	Pregnancy female rats	Miscarriage female rats	Weight of fetus/g((x ± s)	Weight of placenta/g(x ± s)
A	10	1	0.640 ± 0.051	0.409 ± 0.070
B	11	1	0.429 ± 0.0481^(*)^	0.307 ± 0.077^(*)^
C	10	0	0.383 ± 0.801^(*,△)^	0.301 ± 0.076^(*)^
D	10	1	0.487 ± 0.073^(*,△,▲)^	0.338 ± 0.050^(*,△,▲)^
E	10	0	0.491 ± 0.930^(*,△,▲)^	0.327 ± 0.108^(*,△,▲)^

Annotation: *Compared with group A, P<0.05; ^△^Compared with group B, P<0.05; ^▲^ Compared with group C, P<0.05. Group A was the control group. The rats were fed with distilled water and ordinary fodder. Rats in Group B were fed with distilled water containing 75 mg/L NaAsO_2_ and ordinary fodder. Those in Group C, D, and E were fed with distilled water containing 75 mg/L NaAsO_2_ and fodder containing 0.53 mg/kg, 5.3 mg/kg, and 10.6 mg/kg folic acid respectively

### The effect of NaAsO_2_ exposure on fetal heart development and the intervention effect of folic acid

**Fig. 1:**
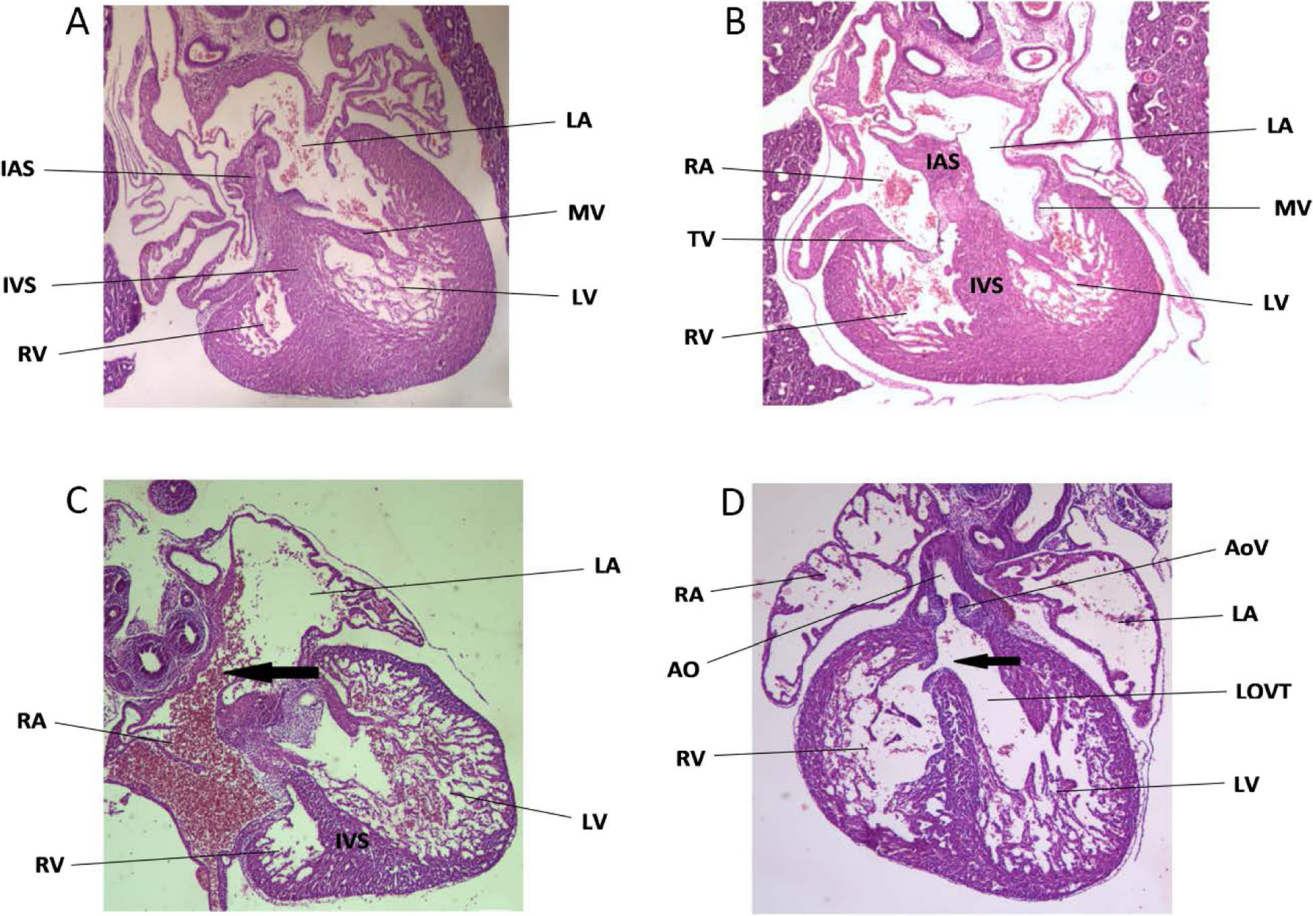
The effect of NaAsO_2_ exposure on fetal heart development during progestation/gestation (×40 times). **a**-**b** pathological section of normal fetal rats heart; **c** ventricular septal defect (↑); **d** atrial septal defect (as arrow pointed) (↑); LV: left ventricular; RV: right ventricular; LA: left atrium; RA: right atrium; IVS: interven tricular septum; IAS: interatrial septum; MV: mitral valve; TV: tricuspid valve;Ao: aorta; AoV: aortic valve; LVOT: left ventricular outflow tract; PA: pulmonary arterial; ↑: anomalous structure

Forty hearts from group A were observed under a microscope, and none of them were found to have cardiac malformations (Fig. 1a and b). In group B, 6 cases of ventricular septal defects (Fig. 1c) and 4 cases of atrial septal defects (Fig. 1d) were found in 44 hearts. In group C, 6 cases of ventricular septal defects and 2 cases of atrial septal defects were found in 40 hearts. In group D, 3 cases of ventricular septal defects and 1 case of an atrial septal defect were found in 44 hearts. In group E, 2 cases of ventricular septal defects and 2 cases of atrial septal defects were found in 44 hearts. The rates of cardiac malformation in the fetal rats from groups A-E were 0, 22.7, 20.0, 9.1 and 9.1%, respectively. The rates of heart malformation in groups B-E was significantly higher than the rate in group A (*P* < 0.05). Groups C-E showed a decreasing trend of fetal heart malformation as the folic acid concentration increased (*P* > 0.05) (Table 2).

**Table 2:** Protective effect of folate on fetal heart development

Group	Number of fetal hearts	VSD	ASD	Amount abnormality rate(%)
A	40	0	0	0 (0%)
B	44	6	4	10 (22.7%)^(*)^
C	40	6	2	8 (20.0%)^(*)^
D	44	3	1	4 (9.1%)^(*)^
E	44	2	2	4 (9.1%)^(*)^

Annotation: ^(*)^ Compared with group A, P<0.05

### The effect of NaAsO_2_ on fetal heart development and the possible mechanism of the protective function of folic acid

**Fig. 2:**
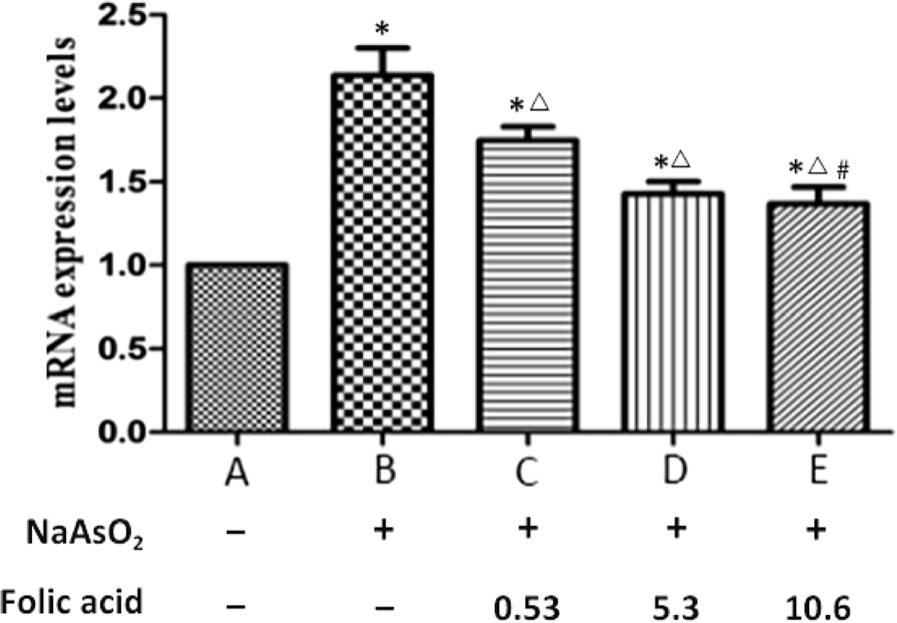
Expression levels of Mef2C in fetal rat hearts. (^*^Compared with group A, *P* < 0.05; ^△^Compared with group B, *P* > 0.05; ^#^Compared with group D, P>0.05)

The PCR results showed that the levels of Mef2C mRNA in cardiomyocytes in groups B-E were significantly higher than those in group A (P<0.05), while the levels of Mef2C mRNA in cardiomyocytes in groups C-E were lower than those in group B (P<0.05). As the folic acid concentration increased, the levels of Mef2C mRNA showed a downward trend, and the difference was statistically significant (*P* < 0.05). However, there was no significant difference between group D and group E (*P* > 0.05) (Fig. 2).

**Fig. 3:**
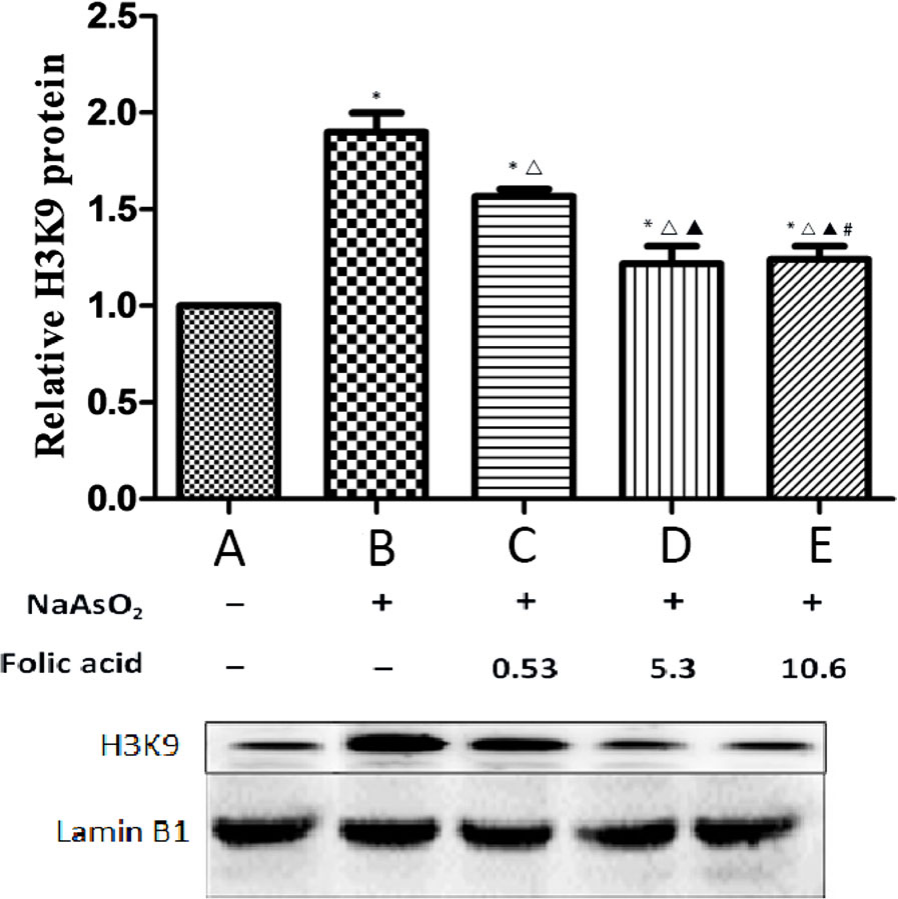
Use Western-blot to test histone H3AcK9 levels in the hearts of fetal rats. (*Compared with group A, P<0.05; ^△^Compared with group B, *P* < 0.05;^▲^Compared with group C, P<0.05; ^#^Compared with group D, P>0.05)

The Western blot results showed that the levels of H3K9 acetylation in cardiomyocytes in groups B, C, D and E were higher than those in group A (P<0.05). The level of H3K9 acetylation in cardiomyocytes in groups C, D and E was lower than that in group B (P<0.05). In addition, as the folate concentration increased, there was a downtrend in H3K9 acetylation, and the difference was statistically significant (*P* < 0.05). However, there was no significant difference between group D and group E (*P* > 0.05) (Fig. 3). The Western blot strip is shown in Fig. 3.

## Discussion

Cardiac development is a complex process, and the sequential activation and correct expression of core cardiac transcription factors are the biological basis for the correct development of the heart [2]. Studies have shown that histone amino acid residues from which the nucleosome protrudes can be modified by acetylation, phosphorylation, methylation, ubiquitination, etc., forming a complex “histone code” and participating in the epigenetic regulation of genetic transcription [15]. As an important regulatory mechanism, histone acetylation plays an important role in the activation and silencing of genetic transcription. As an epigenetic poison, arsenic can cause changes in H3K9 acetylation levels in a variety of cells and tissues. Mef2C is an important core transcription factor in cardiac development and plays an important role in the formation of the ventricular septum [16]. At present, gene sequencing of Mef2C has been completed, and it has been confirmed that in the embryogenesis process in rats, nonsense mutations in Mef2C can cause the heart tube to be unable to form a heart rhythm and can result in disorders in cardiac interstitium development, and a lack of expression of myocardium-related genes [17]. Animal experiments have shown that histone H3K9 acetylation can affect cardiac development by regulating Mef2c gene expression. Insufficient folic acid can cause disorders in intracellular DNA synthesis, cell division and maturation. Insufficient folate can cause dysfunction of intracellular DNA synthesis, cell division and maturation. Animal experiments have confirmed that folic acid intervention in teratogenic factor -exposed mice decreases the risk of CHD in offspring by 16.5% [18]. In this study, SD rats were exposed to NaAsO2 during pregestation/gestation and provided folic acid supplementation. It was observed that NaAsO_2_ caused abnormal heart development in offspring and that folic acid supplementation had a protective effect on embryonic heart development.

The results of this study showed that NaAsO_2_ exposure resulted in a decrease in fetal body weight and placental weight, indicating that NaAsO_2_ can inhibit embryonic growth and development due to embryonic developmental toxicity. The mechanism may be that arsenic and its metabolites are directly toxic to embryonic cells and inhibit cell differentiation and proliferation [19]; it may also be that arsenic and its metabolites lead to placental vascular dysplasia and functional insufficiency [20], which cause embryonic malnutrition, chronic intrauterine hypoxia, and eventually embryonic growth retardation. We observed that the body weight and placental weight of fetal rats increased significantly after folic acid supplementation, suggesting that folic acid can reduce the toxic effects of NaAsO_2_ and promote embryonic growth and development.

The heart is one of the target organs for arsenic toxicity. A large number of studies have confirmed that high arsenic exposure can increase the risk of cardiovascular disease [21], but there is still no solid evidence regarding whether arsenic can cause embryonic heart malformation. The results of this study are consistent with the results of previous experiments. The results showed that VSD and ASD were present in groups B-E, indicating that NaAsO_2_ exposure during pregestation/gestation may cause abnormal morphology in fetuses and increase the risk of CHD. Since events such as atrioventricular development and compartmental separation occur at different stages of the embryonic phase [22], it is thought that NaAsO_2_ may cause damage to multiple structures by interfering with multiple stages of development of the embryonic heart. In this study, it was concluded that folic acid can antagonize the toxic effect of arsenic on the embryonic heart. The results also showed that the levels of H3K9 acetylation in cardiomyocytes in groups C-E were significantly lower than those in group B and that the level of Mef2C mRNA in groups C-E was significantly decreased compared with that in group B. It can be speculated that folic acid may promote the normal development of the heart by interfering with high acetylation levels in cardiac tissue induced by arsenic and regulating the expression of the cardiac development-related gene Mef2C. However, the mechanism by which folic acid decreases the acetylation level of heart tissue remains to be further explored.

## Conclusion

The results of this study showed that folic acid supplementation during the periconception period may protect the embryonic heart by interfering with arsenic-induced high acetylation levels of H3K9 in embryonic myocardial tissues and by regulating the expression of the cardiac development-related gene Mef2C. This will provide a new strategy for the treatment and prevention of congenital heart disease and provide theoretical support for the proper clinical application of folic acid.

## Data Availability

The datasets used and/or analysed during the current study are available from the corresponding author on reasonable request.

## Abbreviations

ASD: Atrial septal defect
CHD: **C**ongenital heart disease
GAPDH: Glyceraldehyde 3-phosphate dehydrogenase
H3K9: Histone H3 lysine 9
HE: Hematoxylin-eosin
Mef2C: Myocyte enhancer factor-2C
NaAsO_2_: Sodium arsenic
NAD: Nicotinamide adenine dinucleotide coenzyme
PVDF: Polyvinylidene fluoride
SIRTs: NAD-dependent histone deacetylase sirtuins
VSD: Ventricular septal defect
